# The success of insulin pump therapy: importance of education of patients and health professionals

**DOI:** 10.3389/fcdhc.2024.1464365

**Published:** 2024-11-14

**Authors:** Bojana Carić, Saša Marin, Jelena Malinović-Pančić, Gabrijela Malešević, Duška Mirnić

**Affiliations:** ^1^ Faculty of Medicine Banja Luka, University of Banja Luka, Banja Luka, Bosnia and Herzegovina; ^2^ Internal Medicine Clinic, Department of Diabetes and Endocrinology, University Clinical Center of Republic of Srpska, Banja Luka, Bosnia and Herzegovina; ^3^ Department of Oral Surgery, Faculty of Medicine, University of Banja Luka, Banja Luka, Bosnia and Herzegovina; ^4^ Ophthalmology Clinic, University Clinical Center of Republic of Srpska, Banja Luka, Bosnia and Herzegovina

**Keywords:** diabetes type 1, education, insulin pumps, bolus calculator, health professionals

## Abstract

The purpose of the study is to investigate the importance of education and re-education for the proper use of the insulin pump (IP) in order to maintain stable glycemic control over an extended period.

**Methods:**

The study was designed as a cross-sectional retrospective study. A total of 168 patients participated in a five-day structured education program in a small group. Following the initial education, 42 patients who met the criteria for continuation of IP treatment continued to be monitored every six months (period I). After six years of follow-up (period II) data from 36 patients were taken and analyzed. The data from the IP were downloaded from the IP Paradigm 754 "VEO" (Medtronic Inc., Illinois, USA) on the personal computer via the CareLink Pro software (Medtronic, Inc., Illinois).

**Results:**

The number of patients using the bolus calculator (BC+) for at least 50% of all administered boluses remained high in both periods. However, BC+ patients statistically significantly increased their A1C value in period II. The average number of hypoglycemias was statistically significantly increased in the group of BC+ patients in period II compared to period I (p=0.009). The continuous glucose monitors (CGM) were used only in period II, so the number of hypoglycemias in period I were roughly estimated.

**Conclusions:**

The long-term success of IP therapy primary depends on the proper use of the device, highlighting the importance of good education and regular re-education for both patients and health professionals. Advanced hybrid technology systems could be particularly in settings with poorly organized healthcare, where re-education is not routinely provided and diabetes control relies heavily on the patient engagement.

## 1 Introduction

Structured diabetes education (SDE) has been a successful therapeutic modality for all patients with type 1 diabetes mellitus (T1DM) on the basal-bolus regimen of intensive insulin therapy (IIT) over the past decades (1). The knowledge gained through SDE helps the everyday challenges of living with diabetes easier (2). Through SDE, patients are trained to adjust the dose of basal insulin and titrate the dose of prandial insulin with the aim of improving glycemic control and enabling them to contribute to glycemic stabilization. This empowers patients to take responsibility for preventing both acute and chronic complications. The success of SDE largely depends on the approach taken by health professionals (1). While there are many educational programs available, some lack proper structure, posing a challenge for effective implementation (3, 4). The literature also highlights the necessity and importance of regular re-education (5). Still, in clinical settings, it is not always feasible.

In recent decades, we have witnessed significant progress in the treatment of T1DM. The introduction of insulin pumps (IP) as a form of IIT and the development of continuous glucose monitoring systems (CGMS) have further improved the quality of life of patients with T1DM. Concurrently, there has been an increased need for patient training in the management of these technical devices (6).

The technological development of systems for CGMS and IP has brought us one step closer to achieving fully automated glycemic management (7, 8). There are clear guidelines for using CGM data in clinical practice (9), as well as recommendations for using automatic insulin delivery systems (AID) (10). There is clear evidence from the real-world evidence (RWE) studies that optimization of glycemic control with the *Advanced Hybrid* Closed Loop (AHCL) systems increases time in range (TIR) and time in tight range (TTIR). In that way, AHCL can improve quality of life and delay or prevent chronic complications of diabetes (7).

Although there is compelling evidence supporting IP therapy over multiple daily insulin injection for T1DM (8, 11), the availability of IP therapy around the world is extremely heterogeneous and is not often related to the country's economic status (12–14). Albeit some high-income counties have a relatively small number of IP and CMGS users, many low-income countries still lack access CGMS, IP therapy and especially AHCL systems (15, 16).

Regardless of the technology progress that reduce the patient's involvement in therapeutic decisions, education remains crucial for the successful initiation and management of IP therapy. Alongside technical education and support, the knowledge of carb counting is the fundamental to the success of IP therapy success, even with AHCL systems (10).

Possible adverse events of IP therapy include diabetic ketoacidosis, skin infections and occlusion of insulin infusion set. However, inadequate education and support could also contribute to unsatisfactory outcomes in glycemic control improvement with diabetes technology (6).

The aim of our retrospective cross-sectional study was to highlight the importance of education for the proper use of the IP to maintain stable glycemic control over a six-year period through the use of a bolus calculator (BC) and other advanced IP options in adult patients with T1DM in the absence of AHCL systems.

## 2 Methods

### 2.1 Study design

The study was designed as a cross-sectional retrospective study. Of the 353 patients screened, 168 patients fulfilled the SED criteria (T1DM, ≥18 years, A1C≥7.5%).

Most of educated patients improved their metabolic control with the knowledge acquired through the educational program. Those who did not achieve good diabetes control were advised to continue treatment with insulin pumps (IP). Due to limited financial resources provided by the Health Insurance Fund, insulin pumps in the Republic of Srpska are still available only through a tender, with a limit of 25 pumps per year. From the initial 168 patients educated from December 2011 to May 2014, 42 patients met the criteria for continuation of IP treatment. Indications for treatment with an insulin pump include preconception, frequent unrecognized hypoglycemia, and incipient diabetic nephropathy. For these patients, achieving better metabolic control was deemed crucial. Insulin pumps were provided from February 2014 to August 2015 at the Department of Endocrinology, University Clinical Center of the Republic of Srpska. Exclusion criteria included advanced microvascular complications and mental impairment.

After the implementation of IP therapy, patients continued to be monitored as part of regular endocrinological examinations every six months. Glycemic control was assessed through A1C and the glycemic profile through SMBG. Additionally, the occurrences and development of complications were also monitored.

During one year follow-up (period I), four patients on IP therapy moved out of the Republic of Srpska, and two patients declined to download data from their IP devices. Thus, the number of patients available for data analysis was reduced to 36.

**Figure 1:**
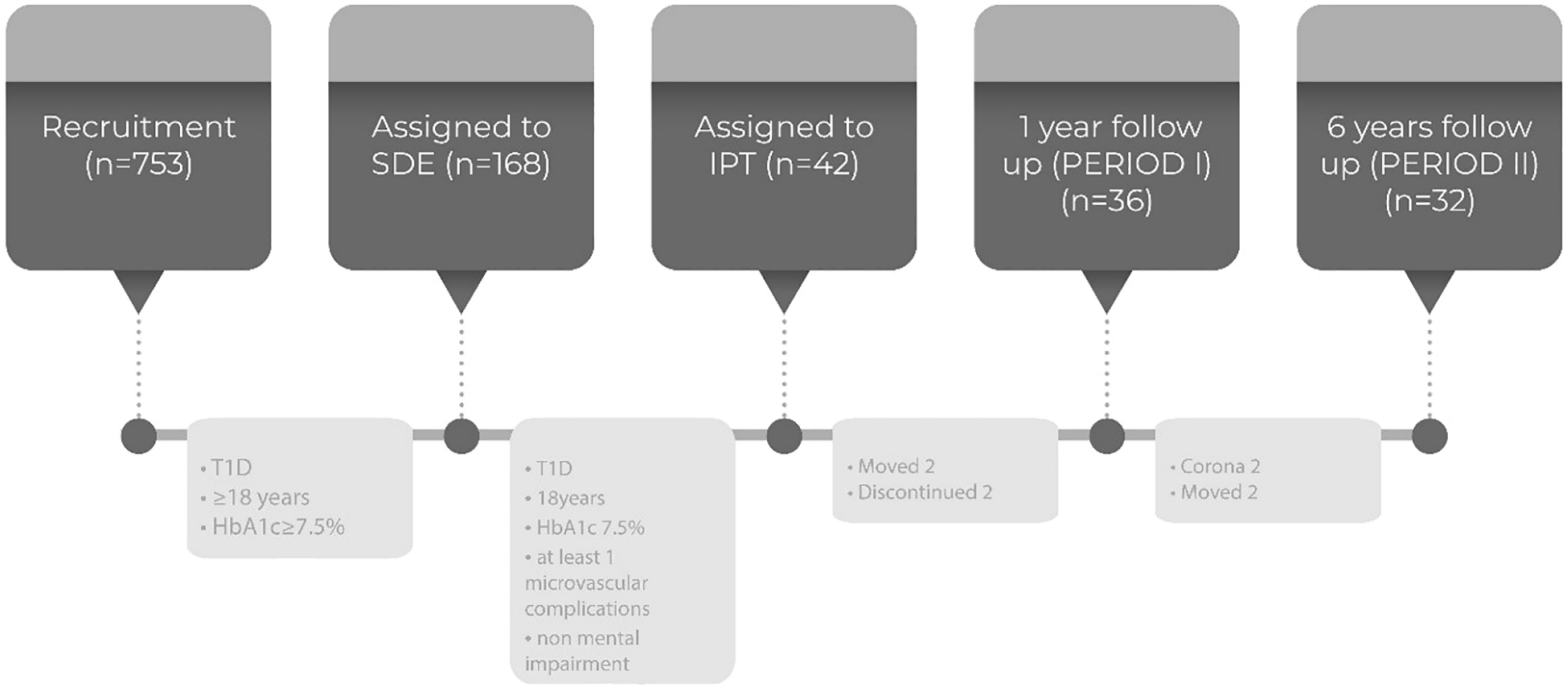
Flow chart of the study.

During six years of follow-up (period II), two additional patients moved out of the Republic of Srpska and two patients died due to COVID-19 infection. Consequently, data were collected for 32 patients in the second period (six years after the initial education) (**Figure 1**).

### 2.2 Education

**Figure 2:**
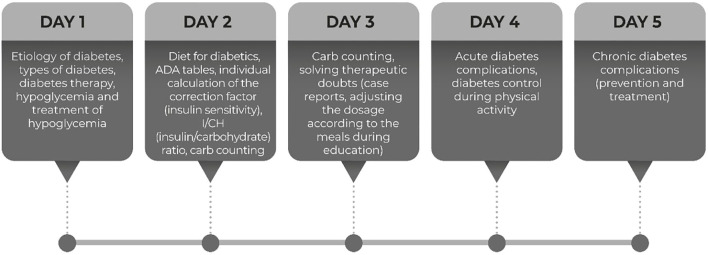
Schedule for five days educational program.

The education was intended for adult patients with T1DM on IIT using basal-bolus regimen who had unsatisfactory glycemic control (A1C > 7.5%) for longer than six months. The education was based on the DAFNE (Dose Adjustment For Normal Eating) program (17, 18). It was conducted for six hours a day, for five consecutive days, at the Department of Endocrinology, in groups of 5-8 patients (**Figure 2**).

The structured education covered the following topics: etiology of diabetes, types of diabetes, diabetes therapy, acute complications of diabetes, diet for diabetes with carb-counting, individual calculation of the insulin sensitivity, carbs ratio and ADA tables, diabetes control during physical activity, and chronic complications of diabetes. The patients were empowered to self-adjust the dose of prandial insulin based on SMBG that promote self-management in everyday life. These educational topics are summarized in the timetable presented in **Figure 2**.

Carb counting was explained using ADA tables, brochures, and educational materials. Patients had two meals daily as part of the hospital education. They learned how to adjust their insulin dose based on measured glycemia and the planned intake of carbohydrates, proteins, and fats, using personal examples.

There were no facilities for performing physical activity within the department. Patients were encouraged to engage in physical activity after the end of the second day of education, in the afternoon, while monitoring glycemia and carbohydrate intake. The effects of physical activity were discussed individually with each patient the following day. As part of the educational program, acute complications, primarily hypoglycemia, were covered for one day. Patients learned how to prevent, recognize, and treat hypoglycemia. They also received guidance on adjusting insulin doses for meals and physical activity through real-life examples involving hypoglycemia.

Within the education section on acute complications, the procedures for sick days, as well as monitoring symptoms and signs of diabetic ketoacidosis and adjusting insulin therapy in the event of ketonuria, are emphasized.

On the last day, the discussion focused on chronic complications, available therapeutic options for treating these complications, and the importance of maintaining good diabetes control to prevent their progression. Patients with poor diabetes control, even without diagnosed complications, also received education. The majority of them continued treatment with the basal-bolus regimen of intensified insulin therapy.

Knowledge about diabetes was tested in writing both at the beginning and at the end of the education. Support with educational material was provided by pharmaceutical company Roche (Switzerland), through its representative office in Bosnia and Herzegovina.

The team of educators comprised three endocrinologists, two nurses, and two nutritionists. One group of patients, over five days, was educated by the same endocrinologist, nurse, and nutritionist. Initially, education was conducted according to a structured education model (**Figure 2**). Unfortunately, due to a lack of staff and insufficient organization, re-education was not carried out systematically. Instead, it was provided only to individual patients with poor diabetes control during regular check-ups by endocrinologists.

The structured education was intended for all patients on the basal-bolus regimen. Patients who did not achieve adequate diabetes control three months after the structured education, and who had indications for continued insulin treatment, underwent technical education on the use of the pump first. This training was conducted by the technical staff from Medtronic representatives. Before adjusting the individual settings for the use of the insulin pump (performed by endocrinologists), patients were educated on how to independently replace the set, use the bolus calculator, adjust the type of bolus to the entered meals, change the injection sites, detect silent occlusions, and follow procedures in the event of allergies or skin infections.

Upon discharge, individual education was provided as needed, with ongoing support both health professionals and technical support staff.

During the observed period, fifteen pumps per year were allocated to patients under 18 years of age, and ten pumps per year were allocated to adult patients. During the COVID-19 pandemic, which falls within the examination period, tenders for insulin pumps were not announced. There were no VEO pump failures during this period. From 2022 onward, MiniMed 640G and MiniMed 740G pumps are available to patients under 18 years of age, while Ypsomed insulin pumps are allocated to adult patients. Over the last two years, adult patients who experienced VEO pump failures have been eligible for treatment with Ypsomed pumps. At the end of 2023, the MiniMed 780G was registered, but the Health Insurance Fund of the Republic of Srpska does not provide reimbursement for this device for either children or adults.

### 2.3 Data collection

The analyzed data were downloaded from the IP Paradigm 754 "VEO" (Medtronic Inc., Illinois, USA) to a personal computer using CareLink Pro software (Medtronic, Inc., Illinois)

After one year of treatment (marked as period I) with the IP Medtronic "754" VEO (Medronic Inc., Illinois), during 2015/2016, data were collected on the use of the manual bolus, bolus with a meal, corrective bolus, bolus given via BC, preferences of BC (sensitivity, I/CH ratio, active insulin time), types of boluses (normal, dual, square), total daily dosage of insulin, bolus and basal ratio, number of basal patterns and carb intake. Data were downloaded using CareLink® Professional 3.0 software (Medtronic, Inc., Illinois) for 36 patients (period I). The analysis included the last nine weeks from the scan date, and this time range was consistent across all patients. A1C values for the observed nine/week period were noted, and preprandial and postprandial glycemic profile was recorded over three consecutive days. All patients on IP therapy had their A1C determined at the Institute for Laboratory Diagnostics of the University Clinical Center of the Republic of Srpska on the Cobas c 501 device, Roche Diagnostics, Switzerland (reference range 4.0-6.2%), and glycemic profiles were registered using the Accu-Chek® Performa, Roche Diagnostics, Switzerland.

After the data analysis, re-education was provided for patients who did not adequately utilize the technical features of the IP. These patients were then monitored two times a year as a part of regular endocrinological examinations. During this period, most patients have started using CGMS due to its affordable price. The emergence of the coronavirus pandemic complicated communication between patients and healthcare professionals for nearly two years. The contact was maintained primarily by phone, mostly for delivering IP consumables, usually without personal interaction.

The same data were downloaded from 32 patients after six years of IP use (period II). Data from the last nine weeks from the IP were analyzed for comparability. Twenty-four of them used CGMS during at least two weeks out of the observed nine-week period.

The implied frequency of using the BC was ≥50% of all administered daily (19). Two patient groups were observed based to the frequency of BC use (BC+ group for patients who used BC at least 50% of all boluses; BC- group for patients who used BC for less than 50% of all boluses).

The A1C values for period I and period II were measured on the same device and the glycemic profile was assessed over three consecutive days using SMBG. The average preprandial and postprandial glycemia values were taken for three monitored days in both periods.

All patients voluntarily agreed to participate in the study and signed an informed consent form (Local ethics committee written approval number 18/4-298/22).

### 2.4 Statistical analysis

For statistical analysis, IBM SPSS Statistics 21.0 software was used. In order to compare the differences in the frequency of observed characteristics between the groups of respondents, the Pearson X2 contingency test was used. The distribution normalcy of the observed characteristics was tested with the Kolmogorov-Smirnov normalcy test. In order to compare the average values of characteristics between the groups of respondents, the Student’s t-test for independent samples was used (observed characteristics that have a normal distribution) and the non-parametric Mann-Whitney U test for independent samples (observed characteristics that do not have a normal distribution). When using the Student’s t-test for independent samples, the F test was used in order to grasp the significance of differences in the variances of observed characteristics. To compare the mean values of average basal and average bolus for the same respondents, the Student's t-test for paired samples was used (because the observed characteristics have a normal distribution). Student's t-test for paired samples was used to compare the mean values of the characteristics at the beginning of therapy and the current mean values of these characteristics for the same respondents (if the observed characteristics have a normal distribution), and the non-parametric Wilcoxon test for paired samples (if the observed characteristics do not have a normal distribution). As statistically significant, all the values in which p <0.05 were used.

## 3 Results

Demographic data for both observation periods are listed in **Table 1**. In both observed periods, females represent the majority of patients (75% in period I; 81.25% in period II). Almost all patients were above 30 years old (91.66% in period I and 93.75% in period II). The average duration of T1DM was over 20 years for both sexes (24 years in period I, 32 years in period II).

**Table 1:** Demographic data on patients after Period I and Period II.

	Period I	Period II
Men	9 (25%)	6 (18.75%)
Women	27 (75%)	26 (81.25%)
Above 30 years of age	33 (91.66%)	30 (93.75%)
Under 30 years of age	3 (8.33%)	2 (6.25%)
The duration of diabetes	24,00 (20,00; 30,00) (13,00 - 47,00)	32,00 (30,00;40,00) (19-55)
Usage of CGMS	0%	75%
BC <50% (n)	50% (18)	40.62% (13)

In our study, the number of patients who used BC (BC+) for at least 50% of all given boluses remained high in both periods (50% of patients in period I, 59.38% of patients in period II) (**Table 1**).

BC+ patients showed a statistically significant increase in their A1C value in period II (A1C 7.15; p=0.027). No statistically significant difference in A1C between the two periods was found in patients who did not use the BC (BC-) (p=0.209). The average number of hypoglycemic events was statistically higher in the BC+ group in period II compared to period I (p=0.009). However, no statistically significant difference in the number of hypoglycemic episodes was found between the two periods in BC-patients. No statistical difference was observed in preprandial and postprandial glycemia by SMBG in the observed glycemic profiles between BC+ and BC- patients (**Table 2**).

**Table 2:** Parameters of glycemic control in both observation periods.

		Period I, Mediana (min-max)	Period II, Mediana (min-max)	p
A1C	BC +	6,65 (5,90, 7,20) (5,2 - 8,6)	7,15 (6,70, 7,70) (5,3 - 9,2)	p = 0,027
	BC -	6,20 (5,70, 7,00) (5,1 - 10,0)	6,75 (6,20, 7,70) (5,4 - 8,6)	p = 0,209
Change in average preprandial glycemia (SMBG)	BC+	6,50 (5,60, 7,27) (4,3 - 9,6)	5,78 (5,27, 7,23) (4,4 - 8,3)	*p = 0,352*
	BC -	5,82 (5,47, 7,43) (4,1 - 8,5)	6,73 (5,40, 6,97) (5,2 - 7,9)	*p = 0,699*
Change in average postprandial glycemia (SMBG)	BC+	0,85 (-0,47, 2,57) (-4,0 - 3,7)	1,65 (1,00, 2,27) (-0,3 - 3,5	*p = 0,151*
	BC-	1,85 (0,73, 2,80) (-1,1 - 4,8)	1,85 (1,33, 2,83) (0,0 - 3,3)	*p = 0,802*
Hypoglycemia/day (n)	BC+	1,50 (1,00, 3,00) (0,0 - 5,0)	3,00 (2,00, 4,00) (1,0 - 5,0)	p = 0,009
	BC-	2,00 (1,00, 4,00) (1,0 - 6,0)	1,50 (1,00, 2,00) (1,0 - 4,0)	p = 0,075

The comparative parameters of IP functions are listed in **Table 3**.

**Table 3:** IP usage data obtained through CareLink Pro software from IP in both observation periods.

		Period I, min-max	Period II, min-max	p
Average number of boluses (n)	BC+	5,50 (4,05, 7,19) (3,1 - 9,4)	5,96 (4,79, 7,16) (3,1 - 11,3)	p = 0,061
	BC-	6,17 (4,48, 7,35) (2,0 - 16,0)	6,59 (3,83, 8,59) (2,6 - 14,2)	p = 0,220
Manual bolus (n)	BC+	0,07 (0,00, 1,00) (0,0 - 3,8)	0,09 (0,00, 0,90) (0,0 - 5,3)	p = 0,937
	BC-	4,63 (2,62, 6,19) (0,5 - 14,3)	4,61 (3,02, 7,88) (1,0 - 12,7)	p = 0,363
Bolus via BC (%)	BC+	4,84 (3,71, 5,57) (2,9 - 8,5)	5,02 (3,63, 6,41) (2,2 - 9,8)	p = 0,080
	BC-	1,43 (0,08, 1,73) (0,0 - 2,8)	1,66 (0,10, 2,04) (0,0 - 2,9)	p = 0,198
Bolus with a meal (n)	BC+	3,12 (2,59, 4,08) (0,1 - 8,2)	3,60 (2,60, 4,30) (1,4 - 6,2)	p = 0,647
	BC-	0,58 (0,00, 1,86) (0,0 - 4,1)	1,00 (0,10, 1,30) (0,0 - 1,8)	p = 0,476
Corrective bolus (n)	BC+	1,62 (0,62, 3,00) (0,2 - 4,8)	1,65 (1,40, 2,40) (0,2 - 4,9)	p = 0,888
	BC-	1,25 (0,00, 2,05) (0,0 - 3,7)	0,60 (0,00, 1,00) (0,0 - 1,6)	p = 0,006
Bolus type "normal" (%)	BC+	100,00 (93,12, 100,00)(62,3 - 100,0)	100,00 (88,47, 100,00)(63,4 - 100,0)	p = 0,074
	BC-	99,54 (97,49, 100,00)(55,6 - 100,0)	100,00 (81,35, 100,00)(46,5 - 100,0)	p = 0,169
Bolus type "dual" (%)	BC+	0,00 (0,00, 3,40) (0,0 - 26,7)	0,00 (0,00, 3,55) (0,0 - 24,5)	p = 0,612
	BC-	0,37 (0,00, 2,40) (0,0 - 44,4)	0,31 (0,00, 1,91) (0,0 - 38,4)	p = 0,263
Bolus type "square" (%)	BC+	0,00 (0,00, 0,00) (0,0 - 11,0)	0,00 (0,00, 0,00) (0,0 - 10,1)	p = 0,655
	BC-	0,00 (0,00, 0,00) (0,0 - 2,2)	0,00 (0,00, 0,00) (0,0 - 1,6)	p = 0,317
Carbs per day (n)	BC+	136,50 (108,00, 207,00)(29,0 - 554,0)	120,50 (78,00, 198,00)(46,0 - 397,0)	p = 0,051
	BC-	51,00 (0,00, 123,00)(0,0 - 184,0)	68,00 (0,00, 118,00)(0,0 - 201,0)	p = 0,220
Total daily dosage of insulin (%)	BC+	35,90 (33,30, 46,40)(17,1 - 72,4)	34,30 (29,50, 51,20)(20,0 - 84,7)	p = 0,456
	BC-	39,45 (32,10, 42,90)(29,3 - 59,0)	40,10 (36,20, 43,50)(30,2 - 63,4)	p = 0,363
Basal ratio (%)	BC+	49,00 (43,00, 52,00)(29,0 - 85,0)	50,50 (46,00, 56,00)(35,0 - 77,0)	p = 0,225
	BC-	54,00 (48,00, 57,00)(38,0 - 69,0)	50,50 (44,00, 52,00)(40,0 - 67,0)	p = 0,002
Bolus ratio (%)	BC+	31,20 (18,20, 48,00) (6,0 - 59,0)	49,50 (44,00, 54,00)(23,0 - 65,0)	p < 0,001
	BC-	45,50 (42,00, 52,00)(31,0 - 62,0)	49,50 (48,00, 56,00)(33,0 - 60,0)	p = 0,001
Basal rates (n)	BC+	4,50 (4,00, 6,00) (3,0 - 8,0)	4,50 (4,00, 6,00) (3,0 - 8,0)	p = 1,000
	BC-	5,00 (4,00, 7,00) (1,0 - 9,0)	5,50 (4,00, 7,00) (3,0 - 7,0)	p = 0,453
Basal pattern (n)	BC+	0,00 (0,00, 1,00) (0,0 - 2,0)	0,00 (0,00, 1,00) (0,0 - 1,0)	p = 0,317
	BC-	0,00 (0,00, 1,00) (0,0 - 2,0)	0,00 (0,00, 1,00) (0,0 - 2,0)	p = 1,000
Active insulin time	BC+	4,00 (4,00, 4,00) (2,0 - 4,0)	4,00 (4,00, 4,00) (3,0 - 5,0)	p = 0,248
	BC-	4,00 (3,00, 4,00) (2,0 - 4,0)	4,00 (4,00, 4,00) (3,0 - 5,0)	p = 0,096
Insulin sensitivity (n)	BC+	1,00 (1,00, 1,00) (1,0 - 2,0)	1,00 (1,00, 1,00) (1,0 - 3,0)	p = 1,000
	BC-	1,00 (1,00, 1,00) (1,0 - 1,0)	1,00 (1,00, 1,00) (1,0 - 2,0)	p = 0,317
I/CH ratio (n)	BC+	1,00 (1,00, 2,00) (1,0 - 3,0)	1,00 (1,00, 2,00) (1,0 - 2,0)	p = 0,655
	BC-	1,00 (1,00, 2,00) (1,0 - 2,0)	1,00 (1,00, 2,00) (1,0 - 2,0)	p = 0,414

Statistically significant differences were found in basal and bolus ratio and number of corrective boluses. BC- patients had a statistically more significant difference in basal rate (p=0.002), with a simultaneous increase of bolus rate in period II (p=0.001). In a group of BC+ patients, there was a statistically significant increase in the bolus rate (p<0.001), while the difference between the basal rate was not observed (p=0.225). Patients who used BC used corrective boluses more often, with a statistically significant difference between the two observed periods (**Table 3**).

Statistically significant differences were not found between period I and period II for the BC+ and BC- group in the following parameters: average bolus numbers, manual boluses, boluses given via BC, boluses with a meal, types of boluses (normal, dual and square bolus), carbs per day, totally daily dosage, basal patterns, active insulin time, sensitivity and I/CH. BC+ patients, as well as BC- patients, daily consume more carbs than recommended, but without statistical difference between the observed periods (BC+ p=0.074; BC- p=0.169) (**Table 3**).

## 4 Discussion

Several studies confirmed the sustained success of good initial SED over time for the patients on a basal-bolus regimen of IIT. ^6^

The long-term success of IP therapy depends on numerous factors, with proper use of the device being paramount. This underscores the importance of good education and regular re-education for both patients and health professionals (20–22).

Moshe P et al. provided precise recommendations for training and educating patients who are initiating AID systems (10). Nevertheless, even the well-educated patients require regular re-education in order to maintain the success of IP treatment (19, 21–23). However, there is a lack of specific recommendations for re-education of patients on IP therapy. It has been showed that despite a thorough education at pump initiation, some patients lacked appropriate knowledge in the event of IP failures. The increased risk of hyperglycemia and diabetic ketoacidosis also highlight the need for patients' re-education (6).

The results of the studies monitoring the effects of re-education, have shown a positive impact of re-education on A1C value (24–27). Regular re-education has reduced the number of hypoglycemic episodes and everyday stress (27) and has also maintained IP user skills (26–28). Our results confirmed the need for ongoing re-education, as the improvement of A1C value after initial education worsened over time in BC+ patients. The unsatisfactory management of BC parameters may be due to lack of re-education. Nixon et al. tracked the glycemic control in IP patients for at least five years and found that a third of patients did not change their glycemic control over time. In this group, inadequate re-education was cited as a possible reason for the lack of improvement (22).

According to the literature, the use of BC decreases the number of hypoglycemic episodes (19, 24). Our study found similar results after initial education. However, in period II we found an increased numbers of hypoglycemia in BC+ patients with a high intake of carbs. These results could be attributed to inadequate adjustment of individual BC settings (26). In both observed periods, different types of boluses were not used appropriately, which could further impact glycemic control. This may be due to insufficient adjustment of the basal insulin rates by health professionals, as the number of basal rates remained unchanged. In both observed periods, only the basic basal pattern was used.

On the other hand, there was no significant change in the difference between preprandial and postprandial glycemia, which supports a stable glycemic profile, regardless of the A1C value. It should be noted that 75% of patients in period II used CGMS at least once (for 14 consecutive days) and that the glycemic profile via SMBG was performed along with CGMS (26). Thus, even occasionally use of CGMS, regardless of BC usage, could compensate the lack of regular re-education of patients on IP (24).

Adjusting AIT to 2-3h improves glycemic control by reducing the number of hypoglycemic episodes (23). In our patients, the AIT was set to 4 hours and remained unchanged over time. This could be explained by the lack of re-education for patients and health professionals, as well as the assessment of glycemic control based solely on A1C values.

Erhmann and colleagues showed that a six-month re-education of patients in a small group encourages patients to use temporary basal and bolus options more frequently, as well as to utilize different basal rate profiles more often (27).

The number of BC+ patients over time in our study did not decrease, and that data correlate with data from the literature (22, 28). The high percentage of BC+ patients over time can be explained by good initial education and duration of diabetes before the introduction of IP therapy. The longer duration of diabetes is one of the possible reasons for persistence in BC use (29, 30). Also, most of our patients were over 30 years old, and women represented the majority, contributing to persistence in using IP (30, 31).

BC- patients had a statistically higher number of corrective boluses, which helped maintain stable glycemic control without increasing the number of hypoglycemic episodes. An increase in the bolus/basal ratio contributes to the improvement of glycemic control (30, 32). In our study, a statistically significant increase in the basal ratio was observed in relation to the two periods for the group of BC patients, while a statistically significant decrease in the bolus ratio was observed at the same time.

It should be noted that in Republic of Srpska, there are no health professionals dedicated solely to IP therapy. During education for IP use, the patients were not advised to self-adjust the BC settings by themselves, but perform it under supervision during regular visits. This could be one of the reasons for registered lack of improvement in glycemic control. Management of IP therapy is part of routine practice, and health professionals are often overloaded. Although technology, such as IP and CGM improved diabetes management, it is clear that technology is not enough what is in line with some research studies. The CGM used together with personalized education can improve glycemic control (33). The lack of time needed for adequate monitoring of patients on IP therapy could be mitigated by the introduction of AHCL, which requires less involvement for both of patients and health professionals. In developing countries, with poor health-care organization where re-education is not feasible and diabetes control relies heavily on patient engagement, AHCL could be highly beneficial (14, 34).

## 5 Conclusion

Education and re-education remain crucial for the proper use of IP and effective diabetes control. Adequate initial education and high patient motivation helped maintain a high percentage of patients using BC. However, other advanced IP options are not used adequately, indicating a need for re-education for patients. Health professionals also require re-education and better organization of daily practice to monitor patients on IP therapy effectively.

## 6 Limitation of study

The study is limited by the small number of patients and the fact that contact between patients and staff was hindered during the coronavirus pandemic. Additionally, the overload of health professionals reduces the time available for adequate control and detailed management of patients on IP therapy.

## Data Availability

The original contributions presented in the study are included in the article/supplementary material. Further inquiries can be directed to the corresponding author.
